# Energetics of Endotoxin Recognition in the Toll-Like Receptor 4 Innate Immune Response

**DOI:** 10.1038/srep17997

**Published:** 2015-12-09

**Authors:** Teresa Paramo, Susana M. Tomasio, Kate L. Irvine, Clare E. Bryant, Peter J. Bond

**Affiliations:** 1 Unilever Centre for Molecular Science Informatics, Department of Chemistry, University of Cambridge, Lensfield Road, Cambridge CB2 1EW, UK; 2 Current Address: Cresset Biomolecular Discovery, New Cambridge House, Bassingbourn Road, Litlington SG8 0SS, UK; 3 Department of Veterinary Medicine, University of Cambridge, Madingley Road, Cambridge CB3 0ES, UK; 4 Bioinformatics Institute (A*STAR), 30 Biopolis Str, #07-01 Matrix, Singapore 138671; 5 Department of Biological Sciences, National University of Singapore, 14 Science Drive 4, 117543 Singapore

## Abstract

Bacterial outer membrane lipopolysaccharide (LPS) potently stimulates the mammalian innate immune system, and can lead to sepsis, the primary cause of death from infections. LPS is sensed by Toll-like receptor 4 (TLR4) in complex with its lipid-binding coreceptor MD-2, but subtle structural variations in LPS can profoundly modulate the response. To better understand the mechanism of LPS-induced stimulation and bacterial evasion, we have calculated the binding affinity to MD-2 of agonistic and antagonistic LPS variants including lipid A, lipid IVa, and synthetic antagonist Eritoran, and provide evidence that the coreceptor is a molecular switch that undergoes ligand-induced conformational changes to appropriately activate or inhibit the receptor complex. The plasticity of the coreceptor binding cavity is shown to be essential for distinguishing between ligands, whilst similar calculations for a model bacterial LPS bilayer reveal the “membrane-like” nature of the protein cavity. The ability to predict the activity of LPS variants should facilitate the rational design of TLR4 therapeutics.

Gram-negative bacteria are surrounded by two membranes, separated by the periplasmic space^1^,^2^. Whilst the inner membrane is composed primarily of simple phospholipids, the outer membrane (OM) also contains lipopolysaccharide (LPS). LPS is a large and complex glycolipid, consisting of a variable O antigen component plus core oligosaccharide, covalently bound to a hydrophobic “anchor” termed lipid A (LPA) that typically contains six acyl tails attached to a phosphorylated β-1′,6-linked glucosamine disaccharide headgroup^3^. LPS imparts the OM with important, therapeutically relevant properties. On the one hand, LPS renders the OM impermeable to large polar molecules, and unusually, to hydrophobic molecules^4^, which is crucial for bacterial survival and a major barrier to antimicrobials and antibiotics^2^,^5^. On the other, LPS is an endotoxin which acts as a potent stimulator of the mammalian innate immune system^6^. An optimal host defence against Gram-negative bacteria is dependent upon successful recognition by Toll-like Receptor (TLR) 4, one of several members of the conserved TLR family which are specialized for recognizing a diverse range of pathogen-associated molecular patterns (PAMPs)^7^. Successful PAMP recognition by a given TLR at the cell surface is thought to involve receptor dimerization, and purported conformational changes across the membrane result in recruitment of adaptor molecules to the Toll-interleukin 1 receptor (TIR) domains within the cytoplasm^8^. The propagation of these activating signals leads to subsequent inflammatory responses^9^.

TLR4 represents a major target for vaccine adjuvants, and conversely, inhibition may help to treat TLR4 over-stimulation in bacterial sepsis^10^. However, rational pharmacological manipulation of the TLR4 system is hampered by the fact that subtle variations in the structure of the bioactive LPA component of LPS can have profound and unpredictable effects upon TLR4 activation. Gram-negative organisms possess diverse strategies for LPA modification that allow them to adapt to their local environment and hence evade immune recognition, whilst the host must be able to distinguish between pathogenic LPS forms and those found in the membranes of commensal bacterial communities^11^. The archetypal TLR4 agonist in all species so far studied is LPA from *E.coli*, yet its biosynthetic precursor lipid IVa (LPIVa) – which only differs by containing four instead of six acyl chains in its lipid A component (Fig. 1A) – is an inhibitor of human TLR4^6^ and has been a candidate for clinical development in sepsis treatment. The synthetic compound Eritoran (also known as E5564) is an investigational drug for treatment of severe sepsis. Eritoran (Erit) also has four acyl chains and is an antagonist in all species examined so far^12^,^13^, but it has a rather different structure to lipid IVa (Fig. 1A).

In contrast with other members of the TLR family, TLR4 does not recognize its PAMPs in isolation; instead, its ectodomain recruits the specialized lipid-binding protein MD-2^14^,^15^,^16^. The two curved β-sheets of MD-2 form a nine-stranded, immunoglobulin-like “β-cup” whose deep cavity is capable of binding a variety of hydrophobic moieties^15^,^16^,^17^ (Fig. 1B). We recently demonstrated that MD-2 can undergo “clamshell-like” motions^18^, consistent with structural studies of other members of the MD-2-related lipid-recognition (ML) domain superfamily^19^, such as the distantly related house dust mite allergen proteins^20^,^21^. In the absence of bound ligand, hydrophobic collapse of MD-2 leads to closure of its cavity and induces flexibility in a key region, the βG-βH loop^18^. However, in the presence of ligand, this loop can exist in “closed” (MD-2c) and “open” (MD-2o) states^15^,^16^,^17^ (Fig. 1B). Clamshell motions may be allosterically transmitted to a phenylalanine residue (Phe126) at the tip of this loop, so that binding of inhibitors such as LPIVa result in transition from the MD-2c to MD-2o conformation, destabilizing the interaction between MD-2 and TLR4 in the active receptor complex. The notion that Phe126 in MD-2 is the molecular switch in endotoxic signalling is strongly supported by NMR studies, revealing that it reorients upon binding metabolically labelled endotoxin^22^. Moreover, a Phe126Ala mutant does not prevent ligand binding, but abolishes receptor signalling^22^,^23^,^24^.

In spite of the progress made in characterizing ligand recognition in the TLR4 system, there is still a lack of information concerning the molecular mechanisms by which ligands are distinguished. According to the Phe126 gating hypothesis, MD-2 may exist in thermodynamic equilibrium between multiple accessible states that are coupled to their inherent affinity for a given ligand (Fig. 1B). LPS agonists will thus tend to be bound to the MD-2c conformation, and stabilize the active receptor complex. Antagonists such as LPIVa will favour MD-2o, inhibiting receptor complex formation and signal transduction, whilst competitively blocking its binding site. Being able to predict these equilibria would facilitate rational design of therapeutics targeted towards TLR4.

Biophysical difficulties associated with characterizing the individual conformational states of MD-2 are exacerbated by the extreme hydrophobicity of LPA, leading to low solubility^25^,^26^ and complex supramolecular phase behaviour^27^,^28^, not to mention the physical and biochemical heterogeneity generally associated with experimental studies of endotoxin^24^,^29^. However, molecular simulations offer a means to bypass such experimental complications, and we adopt such an approach here to study a series of endotoxin binding events to MD-2. We first use an established cellular assay to confirm that LPIVa antagonizes LPS-induced activation of TLR4. We subsequently utilize umbrella sampling (US) calculations to rationalize the agonistic versus antagonistic behaviour of lipid ligands by calculating their potentials of mean force (PMFs) and hence free energy associated with binding to MD-2, in its closed (Δ*G*_*c*_) and open (Δ*G*_*o*_) conformational states (Fig. 1B). We show that Δ*G*_*c*_ is more favourable than Δ*G*_*o*_ in the case of LPA, but that this trend is reversed for LPIVa and Erit; in other words, agonist is biased towards binding to the conformation of MD-2 associated with TLR4 activation, whereas the antagonist preferentially binds to the inactive state. Thus, we provide a clear thermodynamic rationale for the ligand-induced switching mechanism associated with TLR4 regulation by MD-2. The binding preference is shown to be lost under conditions in which the underlying protein dynamics are dampened, confirming the importance of the conformational plasticity of the MD-2 “clamshell” in ligand recognition and signalling. Finally, we demonstrate that the PMF for extraction of a single lipid molecule from a model LPA bilayer is on the order of Δ*G*_*c*_, emphasizing that the protein has evolved to create a specialized, “membrane-like” cavity, providing the necessary sensitivity for the TLR4 system to be effective during the earliest stages of bacterial infection.

## Results

### Cellular TLR4 stimulation is competitively inhibited by LPIVa

We first used HEK293 cells transfected with components of the human TLR4 pathway and a reporter assay to test for pharmacological effects of LPS, LPIVa, or both in combination (Fig. 2). Lipid concentrations were chosen based on previous dose-response analysis of species-dependent TLR4 signalling^30^. Treatment of cells with 10 ng ml^−1^ or 100 ng ml^−1^ LPS led to strong receptor stimulation, compared to the unstimulated control. In contrast, LPIVa at high dose (1 μg ml^−1^) showed no agonist activity. Importantly, the same treatment with LPIVa in the presence of sub-maximal or maximal LPS concentrations permitted complete inhibition of TLR4 signalling. This confirms that LPIVa acts as a dose-dependent antagonist, which does not activate TLR4 but presumably binds to MD-2 in a competitive manner to prevent LPS agonist-induced receptor stimulation, consistent with its reported antiendotoxic activity and previous structural^17^ and pharmacological^31^ analyses.

### Conformational plasticity of active MD-2 supports agonist binding

Having confirmed the activities of agonist versus antagonist at the cellular level, we next sought to establish the molecular basis for differences in both binding and activation/inhibition, by calculating PMFs for ligands binding to MD-2 in its signalling-active and inactive conformations. In Fig. 3A, simulation snapshots associated with the (un)binding of hexa-acylated LPA from the cavity of MD-2 are presented. The corresponding unbiased PMFs were calculated for the closed (MD-2c) and open (MD-2o) forms of MD-2, and are shown in Fig. 4A, with *z* corresponding to the distance between the LPA headgroup and stable β-floor of MD-2. The equilibrium bound *z* position (*z*_*eq*_) for MD-2o and MD-2c lies at ~1.3 nm and ~1.5 nm, respectively. Around *z*_*eq*_, the position of LPA can vary by ~0.2–0.3 nm with a minimal free-energy cost, whilst maintaining key interactions with lipid tail and headgroup. Partial protrusion of the lipid molecule may serve to provide flexibility in recognition when presenting variably modified endotoxin to the TLR4 receptor *in vivo*. In both protein conformations, the bound state was stabilized by burial from solvent of the six acyl tails within the MD-2 hydrophobic cavity. This is evidenced by the extensive interaction surface area observed between protein and LPA, amounting to >20 nm^2^ (Fig. 5A) at *z*_*eq*_. Two or three hydrogen-bonds were initially present in both MD-2 states, formed between polar amino acid sidechains and carbonyl and hydroxyl oxygens of the LPA acyl backbone (Fig. 3A, supplementary Fig. S1A), but most were lost early in the PMF (Fig. 3A, supplementary Fig. S1A). Nevertheless, for MD-2c, reorientation of Lys122, a residue proposed to be essential for endotoxin binding specificity^24^,^32^, enabled maintenance of a salt-bridge between its ε-amino group and 4′-PO_4_ as far as ~1.5 nm beyond *z*_*eq*_ (Fig. 3A).

**Figure 1:**
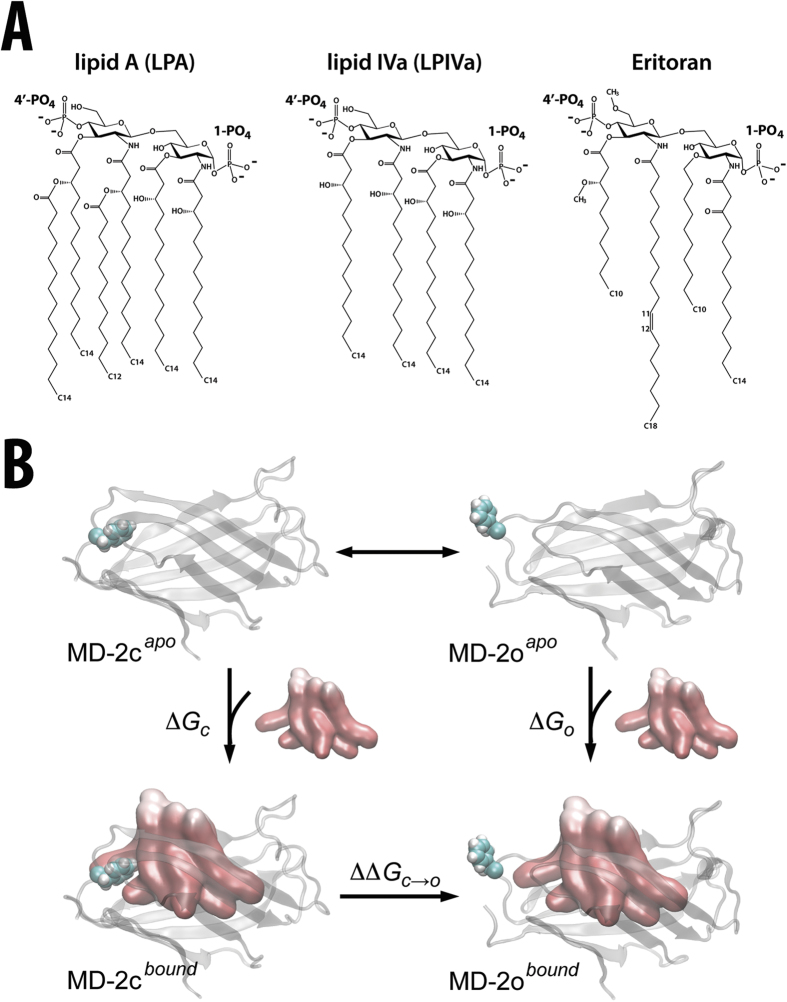
Endotoxic lipid binding to MD-2. **(A)** Chemical structures of lipids employed in this study, a natural TLR4 agonist (lipid A), natural antagonist (lipid IVa), and synthetic antagonist (Eritoran). (**B**) Thermodynamic cycle for ligand binding to MD-2 in its closed, active (MD-2c) and open, inactive (MD-2o) conformational states. Calculation of binding free energies Δ*G*_*c*_ and Δ*G*_*o*_ enables estimation of the equilibrium between the two states of the protein in the presence of a particular ligand (ΔΔ*G*_*c→o*_). Negligible free-energy cost is assumed for ligand-free switching, given the absence of a defined Phe126 conformation during simulation and an RMSD distribution ranging over ~1.5 nm. MD-2 is shown as transparent cartoons, with the Phe126 switch highlighted in spacefill, whilst the lipid A ligand is rendered in molecular surface format.

**Figure 2:**
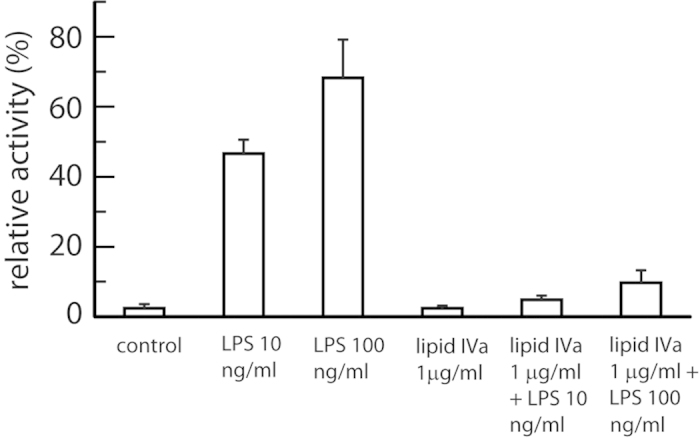
Signaling characteristics of MD-2/TLR4, in cells stimulated with LPS, LPIVa, or both in combination. HEK293 cells were transiently transfected with human TLR4, MD-2 and CD14, together with reporter constructs NF-κB-luc and phRG-TK. Cells were stimulated 48 hours later for 6 hours. Data are from a representative experiment (n = 3 experiments) and expressed as triplicate mean ± SEM for that experiment. LPS activated human TLR4 dose-dependently, whereas LPIVa showed no agonist activity and antagonised LPS at both concentrations of agonist.

**Figure 3:**
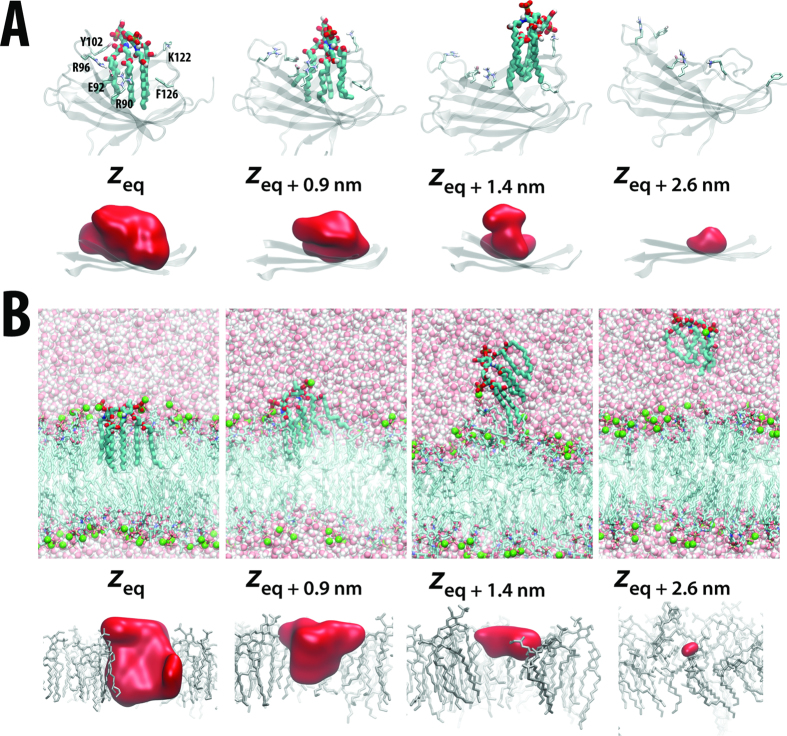
The pathway associated with the PMF for LPA. Snapshots are shown for LPA centred at different positions along *z* with respect to its equilibrium bound position (*z*_*eq*_), with corresponding internal cavity surfaces shown below, for **(A)** the MD-2c protein and **(B)** a symmetric LPA bilayer. The LPA molecule of interest is shown in thick wireframe CPK format. In (**A**) key side chains are labelled and shown in thin wireframe format, with the MD-2 fold rendered as transparent cartoons. In (B) bilayer lipids are represented in thin wireframe format, with Mg^2+^ ions (green) and water molecules (CPK) shown in spacefill representation. Cavities are shown as red molecular surfaces, and represent >50% occupancy calculated using trj_cavity^69^ over the final 5 ns of the corresponding PMF windows.

Beyond *z*_*eq*_, the PMF for each protein conformation exhibits a subsequent smooth rise in free-energy as LPA leaves the binding cavity towards the bulk solvent phase. The hydrophobic tails, which represent the major contribution to the free-energy of binding, exhibit a gradual loss of interaction with protein over a range of ≥1.5 nm. The tails of LPA remain bound to the cavity of MD-2c over a wide region (Fig. 3A) as a result of the “clamshell-like” dynamics of the protein fold as the cavity shrinks (Fig. 5C); the two opposing β-strands close around the partially-buried LPA tails, and enable a cluster of hydrophobic amino acids at the cavity mouth to help shield the lipid tails from external solvent. These include the proposed Phe126 “gate”, along with a series of residues shown to be essential for cellular responsiveness to endotoxin^24^,^33^, including Val82, Met85 Leu87, Phe121, and Tyr131. This adaptation of the MD-2 fold as LPA exits is apparent from the gradual reduction in cavity size, from ~2 nm^3^ at *z*_*eq*_, to <0.5 nm^3^ once LPA is dissolved (Fig. 5B). Over longer distances, LPA gradually leaves the binding cavity (Fig. 5A), and the PMF for MD-2c abruptly plateaus when the lipid finally becomes dissolved within bulk water by ~*z*_*eq+*2nm_. The difference between the PMF at the free-energy minimum and within bulk solvent yields a Δ*G*_*c*_ (Fig. 1B) of ~240 kJ mol^−1^. For the MD-2o conformation, a transition to a local plateau of ~150 kJ mol^−1^ occurs much earlier, at ~*z*_*eq+1nm*_, before undergoing a further transition that begins approximately 0.5 nm further along *z*, yielding a total Δ*G*_*o*_ (Fig. 1B) of ~170 kJ mol^−1^. The local plateau corresponds to earlier exit of the lipid from the open binding cavity leaving the acyl tails to interact only weakly with the protein surface (supplementary Figs S2A and S3A) and hence a reduced capacity for the dynamic cavity to bury the LPA molecule in its open state. In fact, the more open cavity mouth *of MD-2o* already reduces the initial cavity size to ~1.7 nm^3^ at *z*_*eq*_, and subsequently hampers adaptation to the large hydrophobic component of the LPA molecule; instead, there is an abrupt drop in internal volume from ~1.5 nm^3^ to ~0.5 nm^3^ at ~*z*_*eq+1nm*_ as the lipid is expelled from the cavity (Fig. 5B).

Following calculation of Δ*G*_*c*_ and Δ*G*_*o*_, a free-energy cost ΔΔ*G*_*c → o*_ (Fig. 1B) of ~70 kJ mol^−1^ may be estimated for the transition from the closed, signalling-active form of MD-2 to the open, inactive state. Thus, LPA agonist is biased towards binding to the active state of MD-2, as a consequence of the clamshell-like dynamics in the closed conformation that enable the β-cup fold to locally adapt to the bound molecule. To further explore this notion, we again calculated LPA-binding PMFs, but now with the protein fold harmonically restrained to its initial conformation (supplementary Fig. S4A). Under such conditions, a decrease in Δ*G*_*c*_ of ~70 kJ mol^−1^ was observed, and a value of −20 kJ mol^−1^ was estimated for ΔΔ*G*_*c → o*_, thus eliminating the favourable binding of agonist to the active state of MD-2.

### Antagonist biased towards inactive MD-2 binding

**Figure 4:**
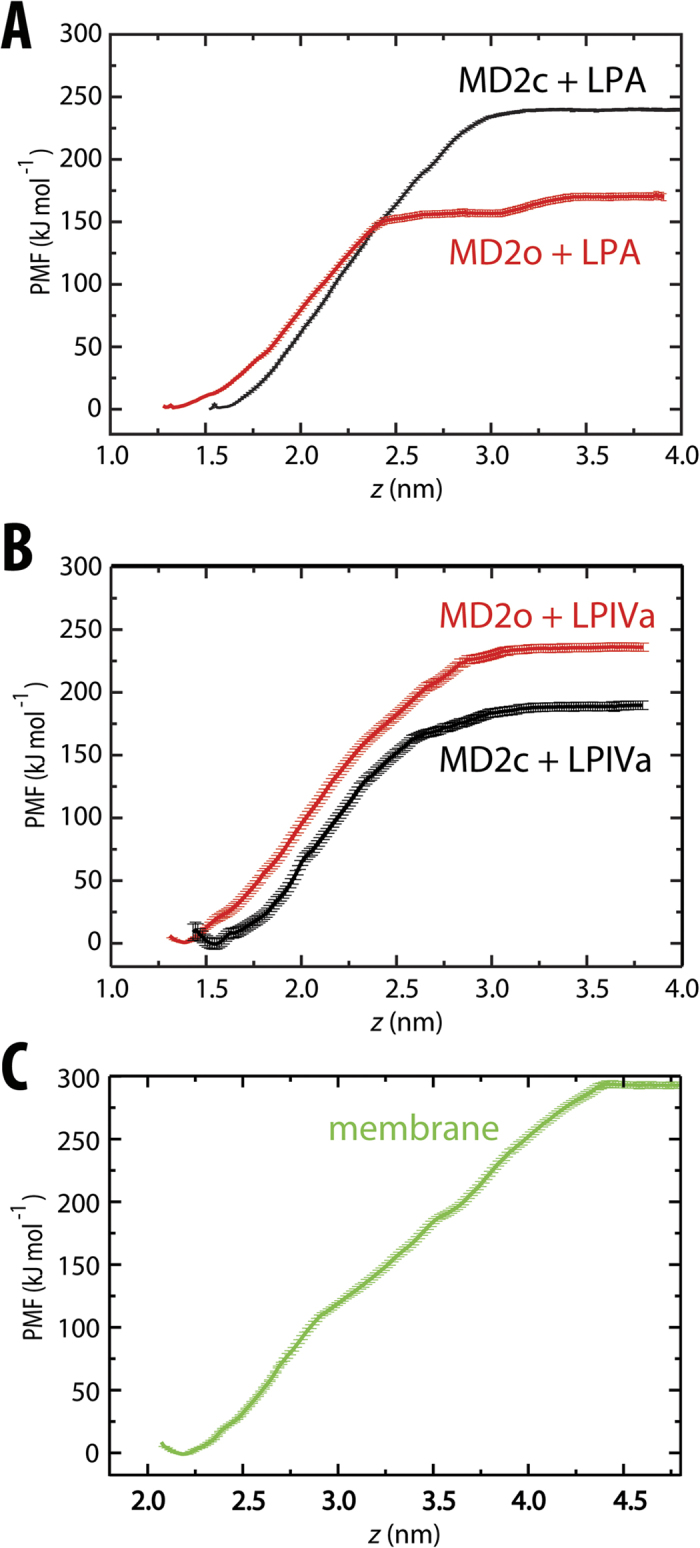
PMF force curves for different lipid molecules calculated as a function of *z*. PMF curves are shown for ligand binding to MD-2c (black lines) and MD-2o (red lines) for **(A)** lipid A agonist, and **(B)** lipid IVa antagonist. The PMF in **(C)** represents lipid A binding to a symmetric bilayer. The centre of the protein or lipid bilayer is at *z* = 0 nm. The PMFs have been normalized so that the minimum lies at 0 kJ mol^−1^. Sample standard deviation was estimated using 200 bootstraps over converged data.

**Figure 5:**
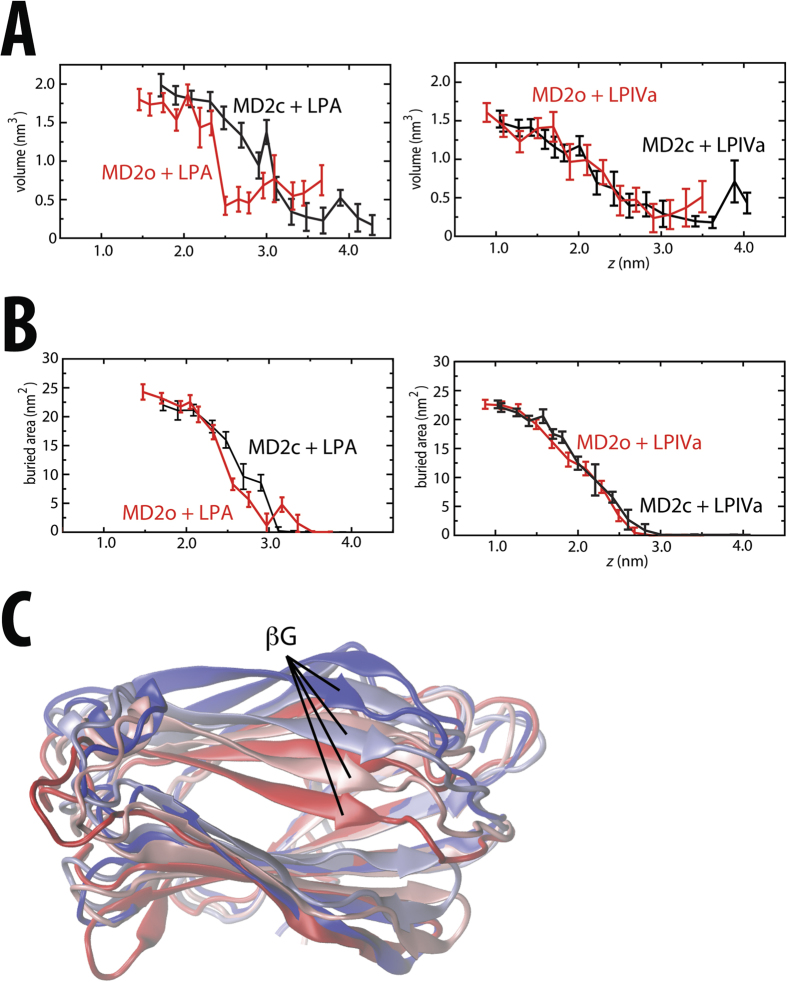
Lipid and cavity behaviour with respect to the binding PMF. **(A)** Protein cavity volume, and **(B)** buried surface area between protein and lipid, calculated as a function of *z* for LPA (top) and LPIVa (bottom), in both the MD-2c (black) and MD-2o (red) conformational states. Cavity volume was measured with trj_cavity^69^. Mean and standard deviations are shown for the final 5 ns of the corresponding PMF windows. **(C)** Snapshots from above the binding cavity surface are overlaid for the MD-2c+LPA system at *z* = 1.7 nm, *z* = 2.7 nm, *z* = 3.1 nm, and *z* = 3.5 nm, colored dark blue, light blue, pink, and red, respectively. The separation between the two opposing β-strands reduces from ~2 nm at to ~1 nm. Protein is shown in cartoons format, with the βG-strand that leads into the Phe126-containing loop labelled.

To test whether antagonist may also be thermodynamically distinguished by different states of MD-2, PMFs were calculated for tetra-acylated LPIVa binding to MD-2c and MD-2o (Fig. 4B). In the equilibrium bound conformation, the MD-2 cavity is already appreciably condensed (Fig. 5A), having adjusted to the smaller volume of LPIVa in comparison with LPA (estimated lipid volumes of 1.3 and 1.7 nm^3^, respectively). The β-strands at the rim of the cavity shrink around the lipid and facilitate the formation of additional hydrogen-bonds in comparison with LPA, between sugar ring hydroxyl and phosphate oxygens, and the sidechains of ionisable residues, including Arg90, Arg96, and Lys122 (supplementary Fig. S3B). However, polar protein-lipid interactions are again rapidly lost prior to *z*_*eq*+*0.5nm*_, for both MD-2 conformations (supplementary Figs S1B and S3B). Subsequently, as for the LPA-bound systems, the PMFs smoothly rise in free-energy as the four acyl tails are removed from the binding cavity (Fig. 5B), before plateauing at ~ *z*_*eq+2nm*_, as LPIVa becomes dissolved (supplementary Fig. S2B).

The size of LPIVa appears to be favored by the (partially collapsed) open state of MD-2 in comparison with LPA, as evidenced by the smoother loss of cavity volume over the entire PMF before plateauing (Fig. 5A). Estimates of ~180 kJ mol^−1^ and ~240 kJ mol^−1^ (Fig. 4B) were obtained for Δ*G*_*c*_ and Δ*G*_*o*_, yielding a ΔΔ*G*_*c→o*_ (Fig. 1B) of around −60 kJ mol^−1^. Thus, a similar magnitude but opposite sign was found in comparison with LPA, confirming that LPIVa acts as a competitive antagonist at the MD-2 cavity that is strongly biased towards binding to the inactive, open state. Again, the conformational plasticity inherent to the β–cup fold appears to be largely responsible for this bias; equivalent PMFs in the absence of protein dynamics reduced ΔΔ*G*_*c→o*_ to −30 kJ mol^−1^ (supplementary Fig. S4B). The residual affinity for the closed-state here is likely due to the protein coordinates being restrained in their equilibrated (i.e. partially collapsed) state.

As a final confirmation of the mechanistic behaviour of MD-2, we also calculated equivalent PMFs for Erit, which has been shown to dose-dependently inhibit LPS-mediated activation of various cell lines and antagonize the toxic effects of LPS in animal models^12^. Unlike the four fully saturated, mono-hydroxylated C_14_ tails of lipid IVa, Erit is less symmetric and includes variably hydroxylated/methylated C_10_ and C_14_ tails, and most strikingly, a long, central, singly-unsaturated C_18_ tail. As shown crystallographically^15^, the double bond in the C_18_ tail has a cis conformation and adopts a 180° turn, effectively yielding five aligned acyl groups within the MD-2 cavity. Thus, despite having a similar number of acyl carbons as LPIVa, the effective volume estimated in the MD-2-bound state is 85% of that of LPA, compared to only ~70% in the case of LPIVa. Once again, a smooth rise in free-energy was observed as the extended acyl tails were removed from the binding cavity, but for both protein conformations the gradual process of exit was extended as a result of the unfolding of the 180° turn in the cis-C_18_ chain (Fig. 1, supplementary Fig. S5). Nevertheless, both ligands had dissolved by ~*z*_*eq+2nm*_, and the free energies Δ*G*_*c*_ and Δ*G*_*o*_ plateaued at ~220 kJ mol^−1^ and ~250 kJ mol^−1^ (supplementary Fig. S5). The high affinity to both states is consistent with the ability of Erit to effectively compete with endotoxin for the MD-2 cavity, but a ΔΔ*G*_*c→o*_ of −30 kJ mol^−1^ again confirms that it should act as an antagonist, biasing the protein towards the inactive state upon binding.

### Membrane-like environment within the MD-2 cavity

**Figure 6:**
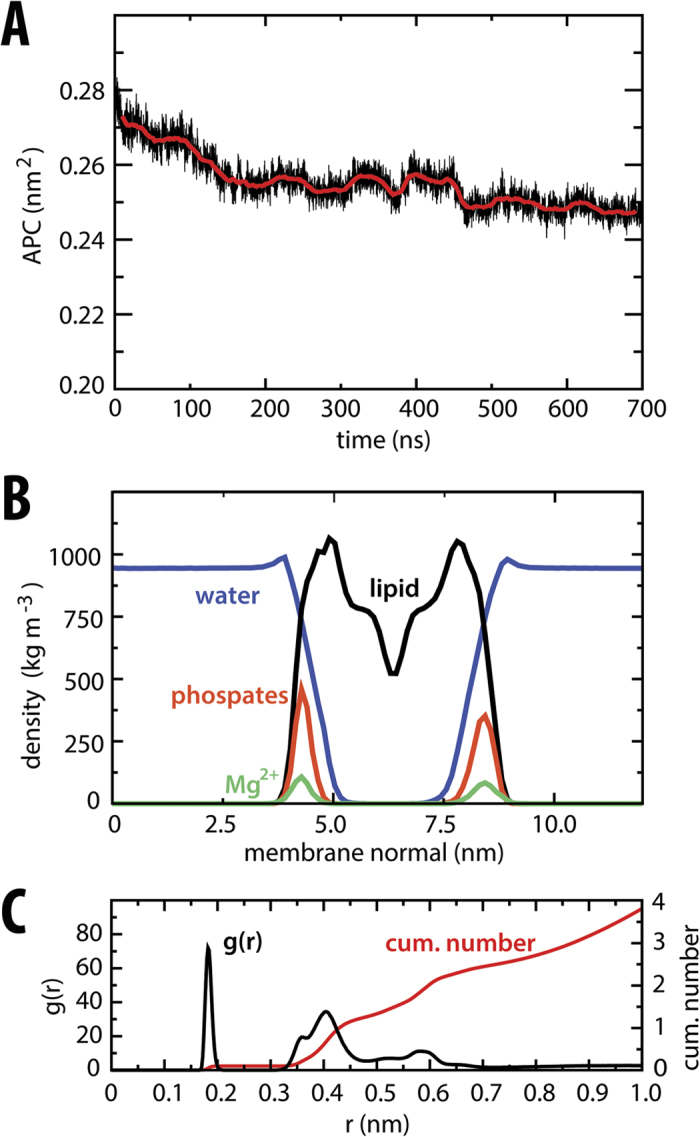
Equilibrium properties of symmetric LPA bilayer. **(A)** Time-dependent area per acyl chain during equilibration of the membrane. **(B)** Density profile of system components calculated over final 200 ns. **(C)** Radial distribution function for LPA phosphate oxygens and Mg^2+^, and corresponding cumulative number of Mg^2+^ ions, calculated over final 200 ns.

Given its extremely low solubility, efficient transfer of endotoxin from bacterial outer membranes necessitates an elaborate relay *in vivo*, including LPS-binding protein (LBP) and CD14^29^,^34^,^35^. It is therefore of interest to establish the thermodynamic cost associated with removal of such lipid from its aggregate microenvironment, prior to TLR4 activation, and to compare this with the energetic gain in its MD-2 bound state. We thus calculated equivalent PMFs for LPA extraction from a symmetric LPA bilayer, after first establishing the model membrane stability. We initially equilibrated a system in the liquid-crystalline phase, in the presence of Mg^2+^ at 323 K, over 500 ns, and then calculated equilibrium properties over a subsequent 200 ns of simulation. This is above the phase transition temperature for free lipid A from *E.coli* of ~315 K^36^, with sufficient water content (<60%) to favour lamellar structures. To ensure the accuracy of the membrane model, we assessed its ability to reproduce certain experimentally-determined structural and dynamic properties in the tensionless *NPT* ensemble. An important measurable property in membranes is the area per acyl chain (AC); from an initial AC value of ~0.28 nm^2^, we observed a gradual reduction over hundreds of nanoseconds, before plateauing to 0.249 ± 0.001 nm^2^ over the final 200 ns (Fig. 6A). This indicates that the AC for the lipid A membrane is significantly smaller than for typical, less tightly packed phospholipid membranes, in agreement with low-angle X-ray diffraction studies for symmetric LPA bilayers^37^ which reported an upper-estimate for the AC of 0.26 nm^2^. This is also close to an AC of 0.251 ± 0.002 nm^2^ estimated using a recent lipid A parameter set developed with the united-atom GROMOS 53A6 force field^38^. Comparable accuracy was also indicated by structural properties along the bilayer-normal, with typical headgroup-to-headgroup separation, deep penetration of water into the hydrophobic core, and overlapping densities for Mg^2+^ ions and phosphorylated headgroups (Fig. 6B), as observed previously in diffraction^39^,^40^ and modelling^38^,^41^,^42^,^43^ studies. On average, one Mg^2+^ ion was found within <0.45 nm of each phosphate group (corresponding to two coordinating “shells”), confirming the close association and tendency for divalent cations to cross-link lipids and stabilize the lamellar phase^42^,^43^ (Fig. 6C). Finally, dynamic properties were also in reasonable agreement with experiment; we measured lateral diffusion coefficients of ~1 × 10^−9^ cm^2^ s^−1^ over the latter half of the membrane simulation, in agreement with fluorescent labelling measurements^44^. Whilst the phosphate and counterions on either leaflet were equally populated, a slight asymmetry in the distributions resulted from the slow sampling of lipid dynamics in the tightly packed and cross-linked LPA bilayer.

The removal of a single 6-tail LPA molecule from the 32-lipid bilayer (equal to a total of 192 acyl chains) during calculation of the PMF represents a potentially substantial disruption to the membrane structure, but the partial lipid density as a function of the bilayer cross-section and global membrane AC remained relatively unperturbed prior to and following LPA extraction (supplementary Fig. S6A,B). Strikingly, the PMF for LPA binding to the bilayer environment was similar to that for LPA binding to MD-2c. Beyond *z*_*eq*_, a relatively smooth rise in free-energy is observed in the PMF, until plateau beyond ~*z*_*eq+2.1*_ nm, once LPA enters the bulk solvent phase (Fig. 4C). The difference between the free-energy values in these two regions indicated an energetic cost for LPA extraction of ~290 kJ mol^−1^. A difference of ~50 kJ mol^−1^ in comparison with the Δ*G*_*c*_ of 240 kJ mol^−1^ for protein is a result of Mg^2+^ salt effects; calculation of an alternative (less biologically relevant) membrane PMF in the presence of Na^+^ counterions yielded a free-energy cost of ~250 kJ mol^−1^ (supplementary Fig. S4C). Divalent cations are able to locally cross-link the membrane-bound headgroup phosphates to those of the extracted lipid (Fig. 3B), up to as far as ~*z*_*eq+1.5*_ nm (supplementary Fig. S7). Intriguingly, this is reminiscent of the salt-bridge formed between phosphate and the essential Lys122^24^,^32^ in MD-2c (Fig. 3A) described above, further supporting its proposed role in providing *specificity* for endotoxin binding.

**Figure 7:**
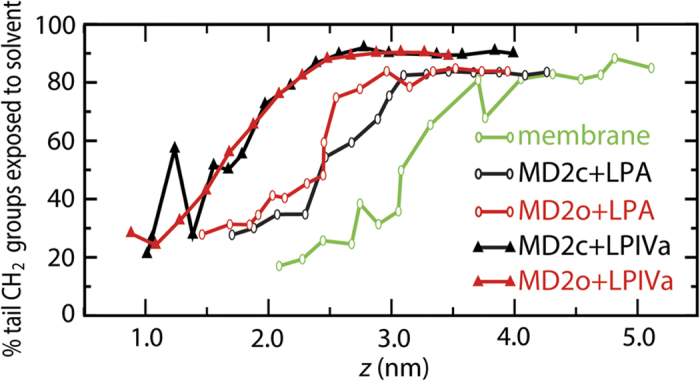
Lipid acyl tail exposure with respect to the binding PMF. The percentage of total acyl tail carbons in LPA and LPIVa that were exposed to solvent is shown as a function of *z*. Solvent-exposed CH_2_ groups are defined as those which make at least one contact (within 0.4 nm) of a water molecule. Means are shown over the final 5 ns. Standard deviations were 30–40% for each data point.

The bulk of the binding free-energy for the membrane again largely results from burial from solvent of almost 20 nm^2^ of surface area associated with the hydrophobic acyl tails (supplementary Fig. S2C). Similarly to the dynamic MD-2 binding pocket, a voluminous “cavity” in the membrane leaflet is gradually lost, becoming filled by surrounding lipid as LPA exits the bilayer (Fig. 3B). Figure 7 shows the percentage of total lipid tail carbons that are exposed to solvent as a function of the *z* coordinate. In the systems containing LPA, a similar pattern is observed, in which the solvent exposure of CH_2_ groups increases by only ~10% over the first ~1 nm along *z*, the exposed surface area resulting from water molecules at the interfacial bilayer region or surrounding the rim of the protein cavity, contacting the upper groups of the acyl chains. Subsequently, a different regime is entered in which the amount of tail exposure to solvent increases more rapidly, prior to plateauing once the lipid molecule is dissolved in solvent. The initial, slow regime has previously been observed for phospholipid bilayers, and was shown to result from elastic deformation of the membrane^45^. Thus, whilst topologically the internal cavities in the equilibrium bound state look rather different between LPA membrane and protein systems (Fig. 3A,B), apparently they create similar local environments that help to “anchor” the acyl tails. This is further supported by the observation that in the bound state of the bilayer or protein (particularly MD-2c) systems, the local endotoxin tail dynamics, as measured by deuterium order parameters, adopt profiles expected for typical membrane systems^38^,^42^ (supplementary Fig. S6C). Thus, the MD-2 cavity appears to favour endotoxin binding by creating an environment that both structurally and dynamically resembles a lipid membrane. In the case of LPIVa, the elastic regime seems less pronounced; for MD-2o, a smaller width of ~0.5 nm along z is associated with slowed CH_2_ exposure, whilst alternation between partially buried and exposed states is apparent for MD-2c prior to complete dissolution. Thus, the MD-2 cavity is apparently less adapted to binding of antagonistic ligands.

## Discussion

Various lines of evidence support the role of Phe126 as part of a molecular switch^18^,^22^ that controls the assembly and hence signalling state of the TLR4 · MD-2 receptor/co-receptor complex^16^. By calculating PMFs for several systems, under various conditions to ensure robustness, we have now provided a thermodynamic rationale for this proposal. LPA is more favourably bound to the conformational state of MD-2 that supports formation of the signalling-active heterotetrameric receptor complex. In contrast, LPIVa and Erit exhibit comparably favourable binding, consistent with their competitive antagonist activity, but to an alternative MD-2 conformation that disfavours receptor complex formation. Whilst the internal cavity of MD-2 is hydrophobic and solvent-buried, generally favouring acyl tail binding, we propose that the extraordinary malleability of the β-cup clamshell provides additional elastic strain energy in a conformation- (and hence switch-) dependent manner. The closed conformation of MD-2 may best match the size, shape and hydrophobicity of hexa-acylated endotoxin, providing additional elastic energy and entropically favoring the ligand upon expansion to the equivalent volume. This is supported by the loss of ~70 kJ mol^−1^ in the PMF when restraining the protein backbone in the MD-2c conformation (Fig S4). In contrast, the open conformation of MD-2, whose binding site is less hydrophobic and more exposed as a result of the reorientation of the Phe126 “plug” from the cavity, appears to favor tetra-acylated ligands, and consistently, a loss of ~20 kJ mol^−1^ in binding energy is now induced when restraining the MD-2o conformation (Fig. S4). Nevertheless, this proposed mechanism awaits experimental support, possibly via e.g. by modulating the dynamics of MD-2 via biochemical cross-linking, and/or by utilizing synthetic ligands with variably unsaturated acyl tails. The dominance of hydrophobic acyl chain burial in the free energy of binding over polar or electrostatic interactions shown here suggests that variations in endotoxin headgroup structure are likely to primarily affect affinity for the complete TLR4/MD-2 receptor complex. This is consistent with recent theoretical^16^ and experimental^46^ data showing that the partial agonistic activity of a mono-phosphorylated lipid A analogue^47^ currently used as an adjuvant in vaccine formulations may cause incomplete signaling via loss of specific electrostatic interactions with TLR4 and an intermediate level of TLR4/MD-2 heterotetramerization. Importantly, such effects would not be picked up using the current approach, which considers only the initial stages of ligand recognition by MD-2.

From a mechanistic point of view, our calculations also suggest that MD-2 creates a membrane-like environment to provide the high affinity necessary to bind endotoxin away from its native bacterial environment, kinetically facilitated *in vivo* by LBP and CD14^29^,^34^,^35^. Accurate experimental estimates of the binding affinity of LPS to proteins (or membranes) are sparse, likely because of its complex structural polymorphism and aggregation behaviour^48^, dependent upon acylation pattern, phosphorylation status, and oligosaccharide heterogeneity. Indeed, the low probability of finding LPS outside of an aggregate phase is highlighted by the <femtomolar concentrations found within the bacterial periplasm^26^, necessitating an elaborate, multi-component trans-envelope Lpt machinery for transfer to the outer membrane at the cell surface^49^,^50^,^51^. Some studies have attempted to measure equilibria between protein and LPS in the presence/absence of serum, but endotoxin molecules are found in various physical and biochemical states, with/without LBP, CD14, diverse lipoproteins, and other lipid or detergent molecules^52^. As such, measurements of interaction with MD-2 are unlikely to reflect a true equilibrium with monomeric endotoxin (or even single CD14/endotoxin complexes)^24^, explaining why tentative estimates of dissociation constants have tended to be variable and in the nanomolar range^14^,^53^. On the other hand, Weiss, Gioannini and co-workers established true equilibria for transfer of hexa-acylated endotoxin between binding sites on MD-2 and CD14, measuring apparent *K*_*d*_ values in the picomolar range, with consistent estimates of CD14/endotoxin concentrations required for half-maximal cell activation^52^. This places an absolute, sub-picomolar upper limit on the true binding activity of free LPS molecules to MD-2, with ever more extreme values expected for LPA in the absence of hydrophilic sugar moieties.

Aggregation experiments with LPS indicated nanomolar critical micelle concentration (CMC) values^25^,^54^ for large aggregate sizes^55^, though these may be underestimated given the complex aggregation behaviour of LPS^27^,^28^,^39^,^42^, whilst reliable CMC values for LPA have not been published so far, due to extreme experimental difficulties of working in the low concentration range of such hydrophobic molecules^25^. Rough estimates based on CMC data available for phospholipids^56^ suggest that a value of well below 10^−10^ M may be assumed for hexaacyl LPA. Tieleman and Marrink^57^, and more recently Pieffet *et al*^45^, calculated PMFs for membrane extraction of the di-acylated phospholipids; as reported here, a smooth drop in the PMF was observed until plateauing upon full dissolution of the lipid into solvent, yielding a total free-energy cost of ~80–100 kJ mol^−1^ in agreement with estimates based on experimentally determined CMCs. This yields a similar free-energy change per tail CH_2_ group to our estimates for LPA binding to a bilayer of ~3–4 kJ mol^−1^, irrespective of changes in simulation conditions, lipid force field, etc. Consistently, experimental models have shown that there tends to be a similar increment for transfer from water into micelle or bilayer for a range of amphipathic surfactants^58^. Strikingly, similar free-energy changes per tail CH_2_ were obtained for *optimal* binding of LPA to MD-2c, suggesting that the energy associated with binding to the protein cavity is comparable to that for aggregation within the membrane phase, in accordance with the high sensitivity of the TLR4 response to invading bacteria *in vivo*. Thus, notwithstanding the experimental difficulties associated with the extreme hydrophobicity of hexa-acylated endotoxin, our estimates for membrane extraction (and MD-2 association) are on the order of what may be expected by extrapolation from both theoretical and experimental models. On the other hand, the optimal binding affinities to MD-2 (i.e. in the open state) for the smaller ligands were similar to those for LPA, meaning that the equivalent free-energy change per acyl CH_2_ group was ~1 kJ mol^−1^ greater. This is likely because of the reduced potential for self-burial of the tails in the case of tetra-acylated compared to hexa-acylated ligands, which effectively makes the CH_2_ groups on average more exposed in solvent, and therefore increases the energetic cost for them to be fully dissolved. Consistent with this, the maximum percentage of solvent-exposed CH_2_ groups is ~10% higher in the case of LPIVa compared to LPA (Fig. 7). The optimal partitioning of large, multi-acylated hydrophobic ligands will depend upon properties such as the surface area is buried upon binding, which is comparable across LPA, LPIVa (Fig. 5), and Erit (Fig. S5), consistent with their similar maximal calculated binding energies, while factors such as lipid shape and volume in solution are also doubtless important.

It has been pointed out that the three-dimensional shape of the LPA component may be correlated with its bioactivity, with “conical” lipid molecules (e.g. LPA) more active than “cylindrical” ones (e.g. LPIVa)^59^. Some hypotheses suggest that the supramolecular structures that arise from different LPA shapes determine their efficiency of delivery to receptors^25^,^48^, and hence indirectly contribute to TLR4 activation, whereas others link the molecular conformation directly to alternative stimulation at the receptor complex recognition site^59^. Our results help to explain why these effects are difficult to separate. On the one hand, it is clear that there is a significant energetic barrier to endotoxin extraction from its aggregated form, which will certainly depend upon the nature of the lipid phase. But equally, the conformational plasticity of MD-2 provides an elegant mechanism for creating a membrane-like environment for stably binding-yet allosterically distinguishing between - lipids with different conformations. Our results emphasize some of the difficulties in using standard structure-based approaches to design molecules for pharmacological manipulation of the TLR4 system, given the need to consider ligand-dependent equilibria between multiple protein conformational states. Further insights into the structural and thermodynamic basis for endotoxin recognition should undoubtedly be gained by taking similar approaches to other ligands of biological and pharmacological interest, as well as by considering other components of the increasingly well-understood TLR4 pathway.

## Methods

### Simulation Details

All simulations were performed using GROMACS 4.5^60^. The CHARMM22/CMAP all-atom force field^61^,^62^ was used to represent the protein, compatible lipid parameters for LPA, LPIVa, and Erit were taken from^18^, and all systems used were explicitly solvated with the TIP3P water model. All simulations were performed in the NpT ensemble, at a temperature of 298 K (for protein systems) or 323 K (for lipid systems), and a pressure of 1 atm. Temperature and pressure were controlled using the velocity-rescale thermostat^63^ and the Parrinello-Rahman barostat^64^,^65^, respectively. Protein and membrane systems used isotropic or semiisotropic pressure coupling, respectively. Equations of motion were integrated using the leapfrog method with a 2 fs time step, and the LINCS algorithm was used to constrain bond lengths^66^. Non-bonded pairlists were generated every 10 steps using a distance cutoff of 1.4 nm. A cutoff of 1.2 nm was used for Lennard-Jones (excluding scaled 1–4) interactions, which were smoothly switched off between 1 nm and 1.2 nm. A dispersion correction was applied to account for truncation of van der Waal’s terms in the protein systems. Electrostatic interactions were computed using the Particle-Mesh-Ewald algorithm^67^ with a real-space cutoff of 1.2 nm. Simulation analysis was performed using GROMACS (http://www.gromacs.org) and VMD^68^. Characterization of the time-dependent shape of the maximal MD-2 binding cavity was performed using trj_cavity using default options^69^. Unless otherwise stated, averages/standard deviations were calculated over the last 5 ns of each PMF.

### PMF Calculations

During US, a harmonic potential with force constant of 1000 kJ mol^−1^ nm^−2^ was applied to the *z*-axis component of the distance between the centre of mass of the diglucosamine headgroup of a single lipid molecule and the centre of mass of a defined reference group. During calculations in the presence of MD-2, the reference group was chosen to be the Cα atoms of the most stable part of the β-cup fold, assessed by calculating the structural drift during 100 ns equilibration simulations. During these simulations, a root-mean-square deviation (RMSD) of ≤0.05 nm was observed throughout, independent of starting protein conformation or ligand-bound state. For the LPA membrane systems, the PMF reference group was chosen to be the heavy atoms of the lipid bilayer. US windows were generated using a series of 5–10 ns steered molecular dynamics (SMD) simulations, in which the lipid was pulled from protein or membrane to their window location using a spring constant of 1000 kJ mol^−1^ nm^−2^ and a pull rate of 0.5 nm ns^−1^. Slower rates resulted in comparable trajectories and similar force vs time curves (supplementary Fig. S8A,B), whilst partial PMFs whose starting coordinates were obtained by a simple process of translation of lipid coordinates resembled those obtained via SMD. In the MD-2/ligand systems, all US windows entailed an initial 0.1 ns of equilibration with position restraints on all non-solvent heavy atoms. For all LPA membrane US windows, 0.1 ns of unrestrained equilibration was performed. An initial default spacing of 0.2 nm was chosen between successive US windows. Additional 0.05–0.1 nm spaced windows were subsequently included as necessary per PMF, to ensure histogram overlap (supplementary Fig. S8E), particularly near to the equilibrium lipid-bound position, resulting in 20–30 windows over a total ~4 nm width along *z*. US simulations were carried out for a minimum of 10 ns, and up to 30 ns per window to ensure PMF convergence, with the final half retained for calculation of free-energy curves and other *z*-dependent properties. PMFs constructed from the US probability distributions were unbiased using the weighted histogram analysis method (WHAM)^70^ with a relative tolerance of 10^−6^. The estimation of the sample error in the calculation of the PMF was then performed using the Bayesian bootstrap method implemented in g_wham, using 200 bootstraps (supplementary Fig. S8C). Convergence was further assessed via block analysis (supplementary Fig. S8D), and in general, windows were considered to have converged when the estimated error over a block of at least 5 ns was under 10 kJ mol^−1^.

### System Setup

The human MD-2c and MD-2o systems were respectively obtained from the crystal structures of the active TLR4 · MD-2 receptor complex bound to LPS^16^ (pdb: 3FXI) and isolated MD-2 bound to LPIVa^17^ (pdb: 2E59). The corresponding “opposite” MD-2 configurations were obtained via pair-wise STAMP structural alignment^71^ and retention of ligand coordinates, followed by a 100 ns equilibrium simulation under the conditions detailed above (supplementary Fig. S9). For each of the four MD-2 systems (closed or open MD-2 conformation, bound to LPIVa, LPA, or Eritoran), initial configurations for SMD and US were based on final snapshots from those preliminary 100 ns equilibration simulations^18^. The symmetric LPA bilayer was equilibrated over 0.7 μs and the final snapshot was used in subsequent US calculations. A periodic cubic box was used for all systems, of sufficient size to ensure that a minimum distance of 1.5 nm was present between protein/endotoxin molecule and the box edges in all sampling windows. All ionisable protein residues were assigned their default ionization states, assuming neutral pH conditions, and sodium/chloride ions were added to neutralize any net charge in the system. Each system was solvated via superposition of a pre-equilibrated box of water molecules. Protein systems were of dimensions ~6 × 6 × 12 nm^3^, containing ~15,000 water molecules and ~50,000 atoms. Membrane systems were of dimensions ~5x5 × 12 nm^3^, with 32 LPA molecules, ~7,000 waters and ~30,000 atoms. Before and after solvation, energy minimization was performed using the steepest descent algorithm in order to relax any undesirable steric clashes between solvent, lipid, and/or protein. Prior to SMD calculations, a solvent equilibration phase was carried out, during which the positions of lipid and/or protein heavy atoms were gradually released from their initial configuration over 1.5 ns of simulation.

### Cell Assays

HEK293 cells were maintained in Dulbecco’s Modified Eagle’s medium (Sigma) containing 10% foetal calf serum (FCS; HyClone), 2mM l-glutamine, 100 iu/ml penicillin and 100 μg/ml streptomycin (referred to as complete culture medium). Cells were plated onto a 96 well plate at 3 × 10^4^ cells/well and transiently transfected two days later. Plasmid-cDNA, together with the reporter vectors pNF-κB-luc (Clontech), encoding a firefly luciferase under an NF-κB promoter, and phRG-TK (Promega), encoding a constitutively expressed *Renilla* luciferase, were transfected into the cells using jetPEI (Polyplus) according to the manufacturer’s instructions. Old medium was removed from plated cells and replaced with plasmid mixtures, together with complete medium. Plasmid-cDNA amounts per well were as follows: human TLR4-pcDNA3 1 ng, human MD-2-pEFIRES 1 ng, human CD14-pcDNA3 1 ng, pNF-κB-luc 10 ng and phRG-TK 5 ng; since 100 ng DNA is recommended for jetPEI, 82 ng empty pcDNA3 was also added. Cells were stimulated two days after transfection. LPS from *E. coli* O157:B8 (Sigma) and LPIVa (Pepta Nova), solubilised in sterile endotoxin-free water (Sigma), were diluted in Dulbecco’s Modified Eagle’s medium containing 0.1% FCS, 2 mM l-glutamine, 100 iu/ml penicillin and 100 μg/ml streptomycin (referred to as 0.1% FCSM). Medium containing transfection reagent was removed from cells and replaced with one of 0.1% FCSM alone, LPS, LPIVa, or LPIVa + ECLPS, and cells incubated for 6 hours at 37 °C/5% CO2. Media were then removed and cells washed with 200 μl warmed PBS. Diluted Passive Lysis Buffer (PLB; Promega) was added at 50 μl per well and luciferase activity determined using the Dual-Luciferase Assay kit (Promega) according to the manufacturer’s instructions.

## Additional Information

**How to cite this article**: Paramo, T. *et al.* Energetics of Endotoxin Recognition in the Toll-Like Receptor 4 Innate Immune Response. *Sci. Rep.* **5**, 17997; doi: 10.1038/srep17997 (2015).

## Supplementary Material

Supplementary Information
