# Supplementary material for: Energetics of Endotoxin Recognition in the Toll-Like Receptor 4 Innate Immune Response
Source: Sci Rep. 2015 Dec 9;5:17997. doi: 10.1038/srep17997 (PMC4673606; doi:10.1038/srep17997)
Supplement: Supplementary Information [file srep17997-s1.pdf]

Energetics of Endotoxin Recognition in the Toll-Like Receptor 4 Innate Immune Response

# - Supplementary Data.

Teresa Paramo[1], Susana M. Tomasio[1,2], Kate L. Irvine[3], Clare E. Bryant[3], & Peter J. Bond[4,5]*

[1] Unilever Centre for Molecular Science Informatics, Department of Chemistry, University of Cambridge, Lensfield Road, Cambridge CB2 1EW, UK

[2] Current Address: Cresset Biomolecular Discovery, New Cambridge House, Bassingbourn Road, Litlington SG8 0SS, UK

[3] Department of Veterinary Medicine, University of Cambridge, Madingley Road, Cambridge CB3 0ES, UK

[4] Bioinformatics Institute (A*STAR), 30 Biopolis Str, #07-01 Matrix, Singapore 138671.

[5] Department of Biological Sciences, National University of Singapore, 14 Science Drive 4, 117543 Singapore.

* To whom correspondence should be addressed: Peter J. Bond, Bioinformatics Institute (A*STAR), 30 Biopolis Str, #07-01 Matrix, Singapore 138671; Tel: +65 6478 8378; E-mail: peterjb@bii.a-star.edu.sg.

Running title: ***Energetic basis for endotoxin recognition.***

September 24, 2015

Supplementary Figures

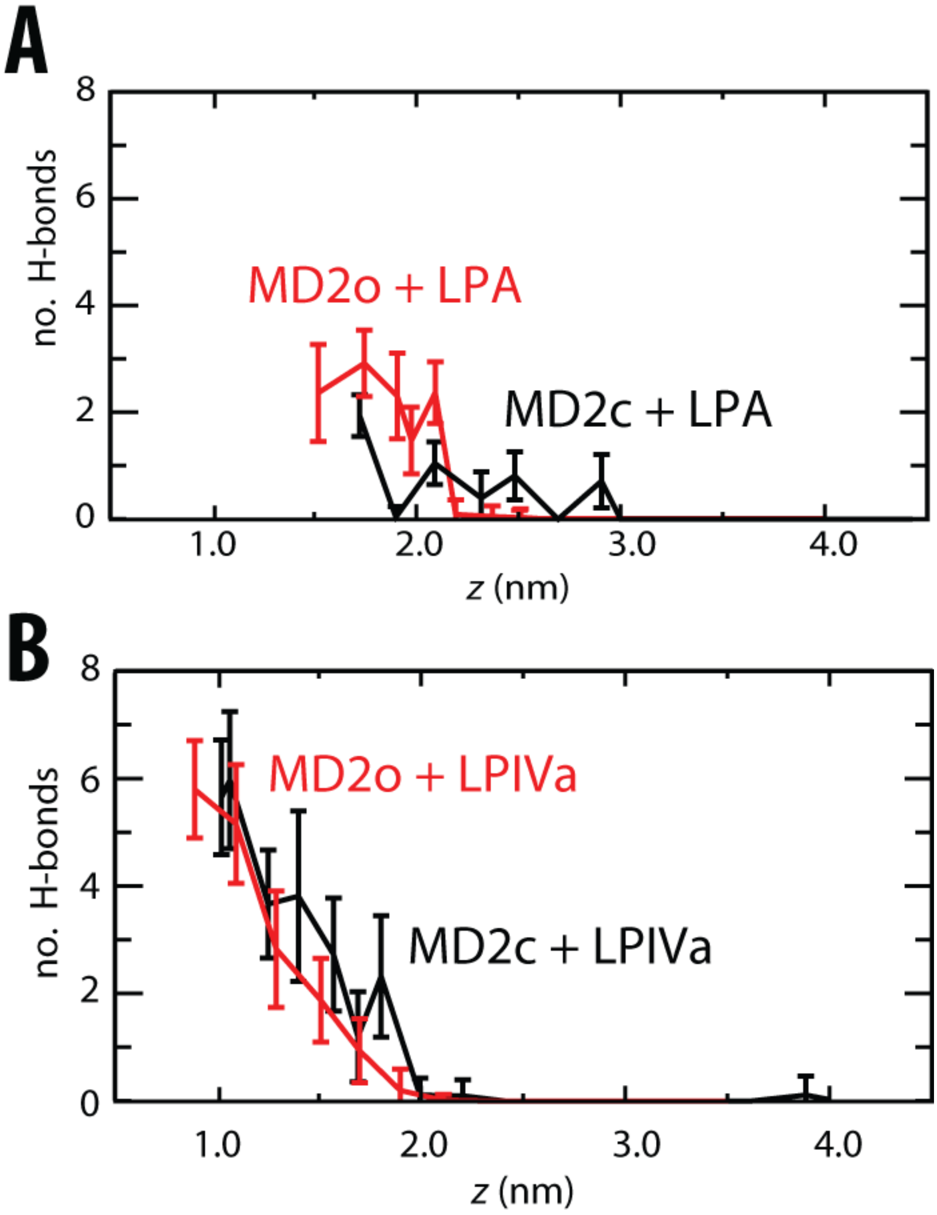

Figure S1. Hydrogen-bond interactions with respect to the binding PMF. Mean number of hydrogen-bonds between lipid and protein are shown as a function of *z* are for **(A)** LPA and **(B)** LPIVa, in both the MD-2c (black) and MD-2o (red) conformational states. Mean and standard deviations are shown for the final 5 ns of the corresponding PMF windows.

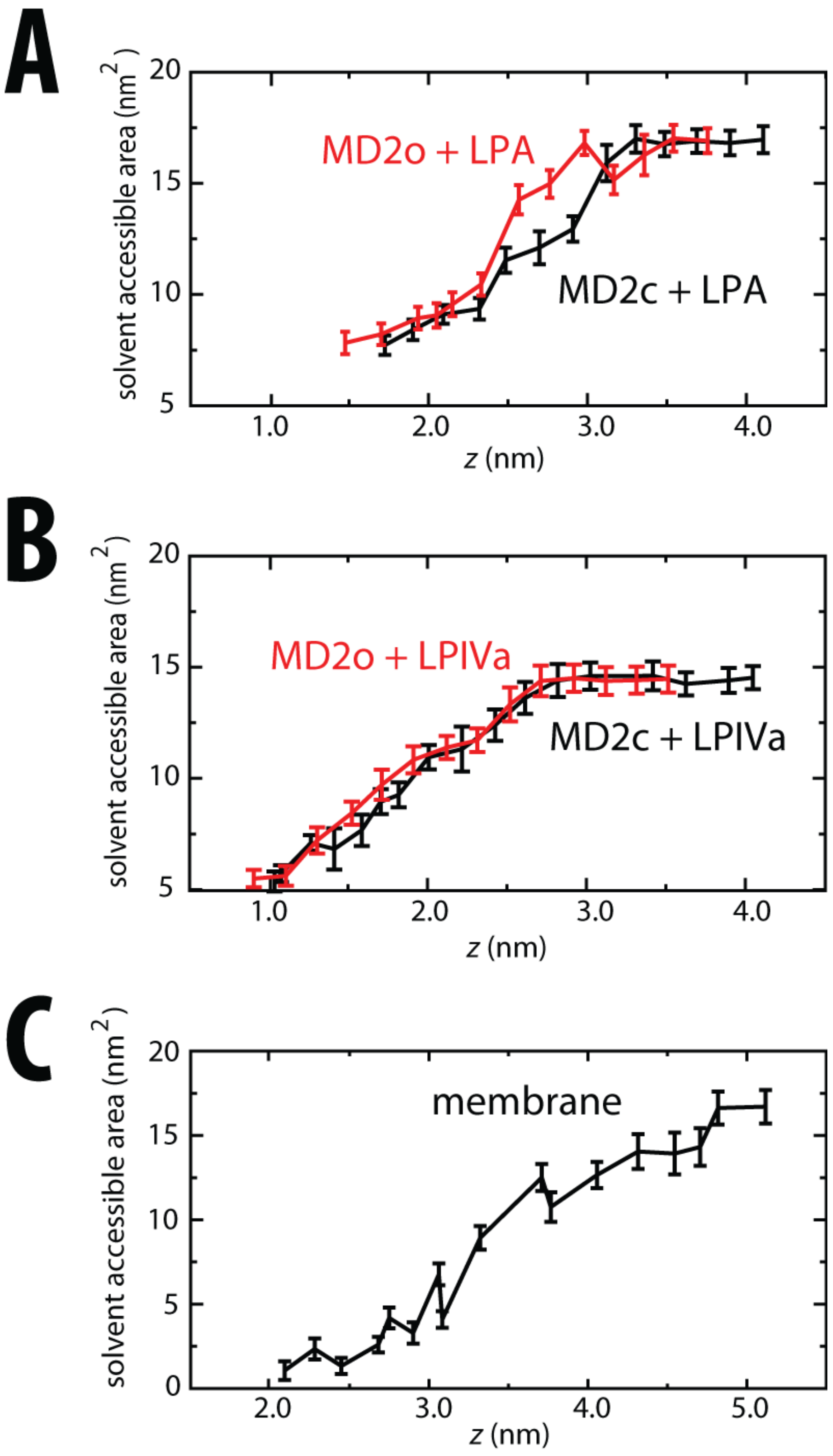

Figure S2. Solvent accessible area of single lipid molecule as a function of PMF *z*. Data are shown for **(A)** LPA bound to MD-2c (black) and MD-2o (red), **(B)** LPIVa bound to MD-2c (black) and MD-2o (red), and **(C)** LPA bound to membrane. Means and standard deviations are shown for the final 5 ns of the corresponding PMF windows.

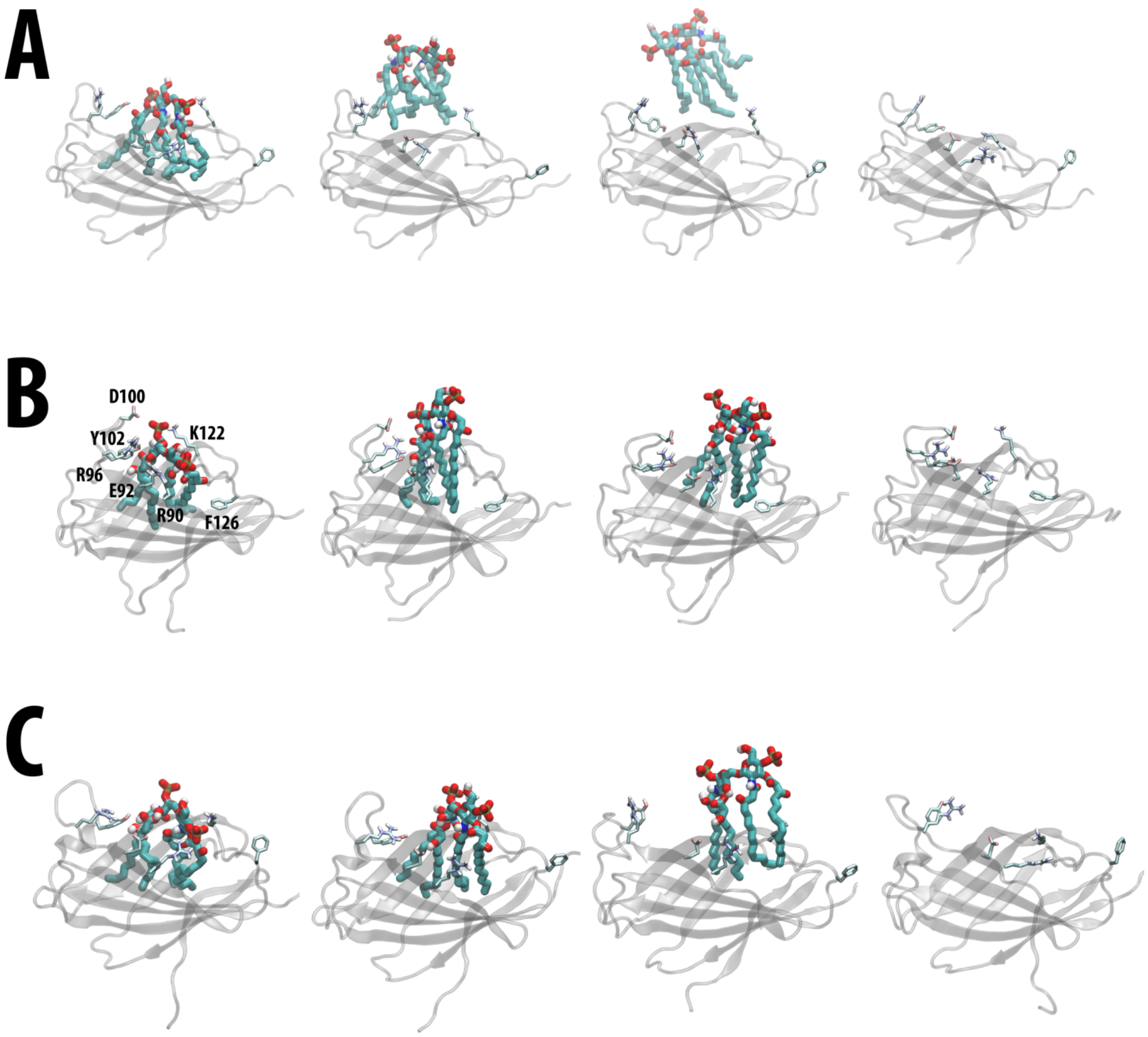

Figure S3. The pathway associated with lipid potentials of mean force binding to MD-2. Snapshots are shown for lipid centred at different positions along z with respect to its equilibrium bound position (z_{eq}), as in Fig 2A, for **(A)** MD-2o + LPA, **(B)** MD-2c + LPIVa, and **(C)** MD-2o + LPIVa. The lipid molecule is shown in thick wireframe CPK format. Key side chains are labelled and shown in thin wireframe format, with the MD-2 fold rendered as transparent cartoons.

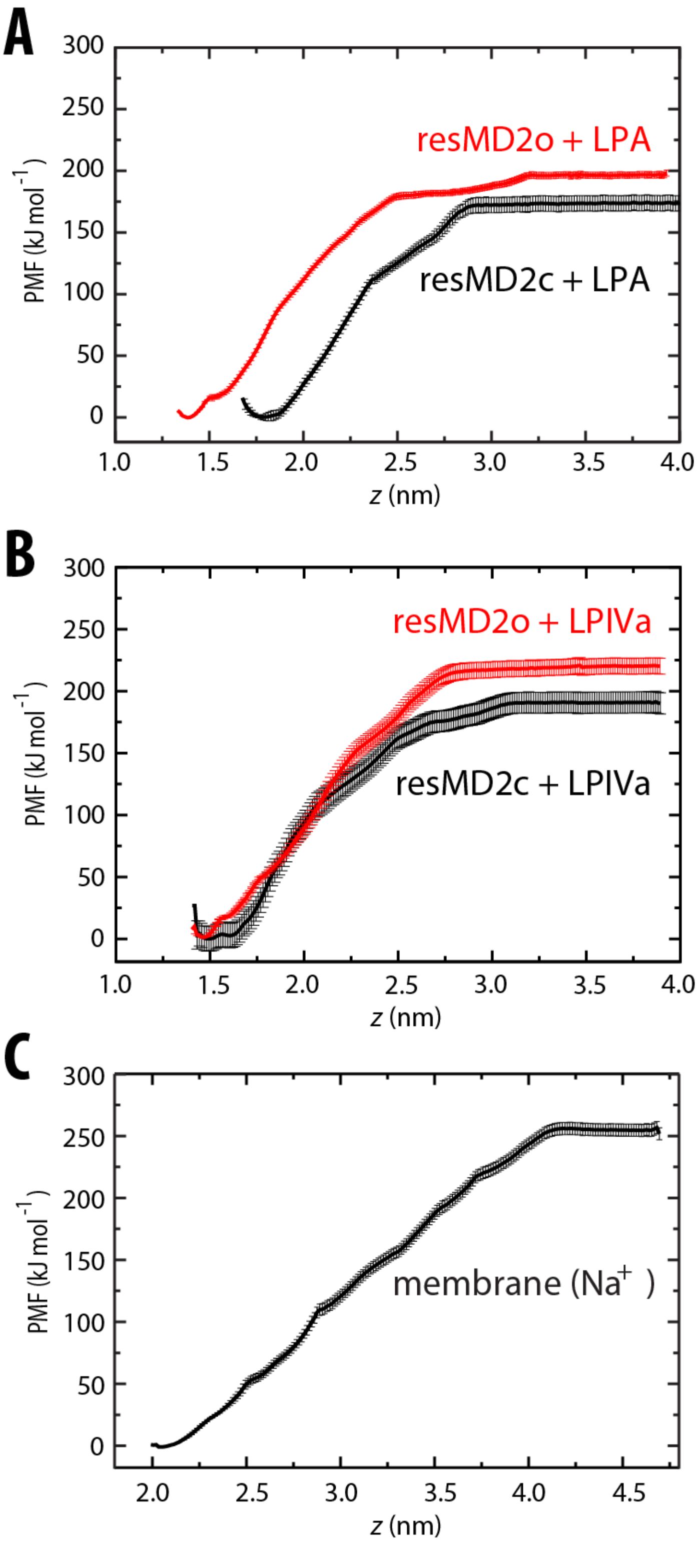

Figure S4. Alternative potential of mean force curves for different lipid molecules calculated as a function of *z*. PMF curves are shown for ligand binding to protein-restrained MD-2c (black lines) and MD-2o (red lines) for **(A)** LPA agonist, and **(B)** LPIVa antagonist. The PMF in **(C)** represents lipid A binding to a symmetric LPA bilayer in the presence of Na^+ counterions. The centre of the protein or lipid bilayer is at $z = 0$ nm. The PMFs have been normalized so that the minimum lies at 0 kJ mol^{-1}.

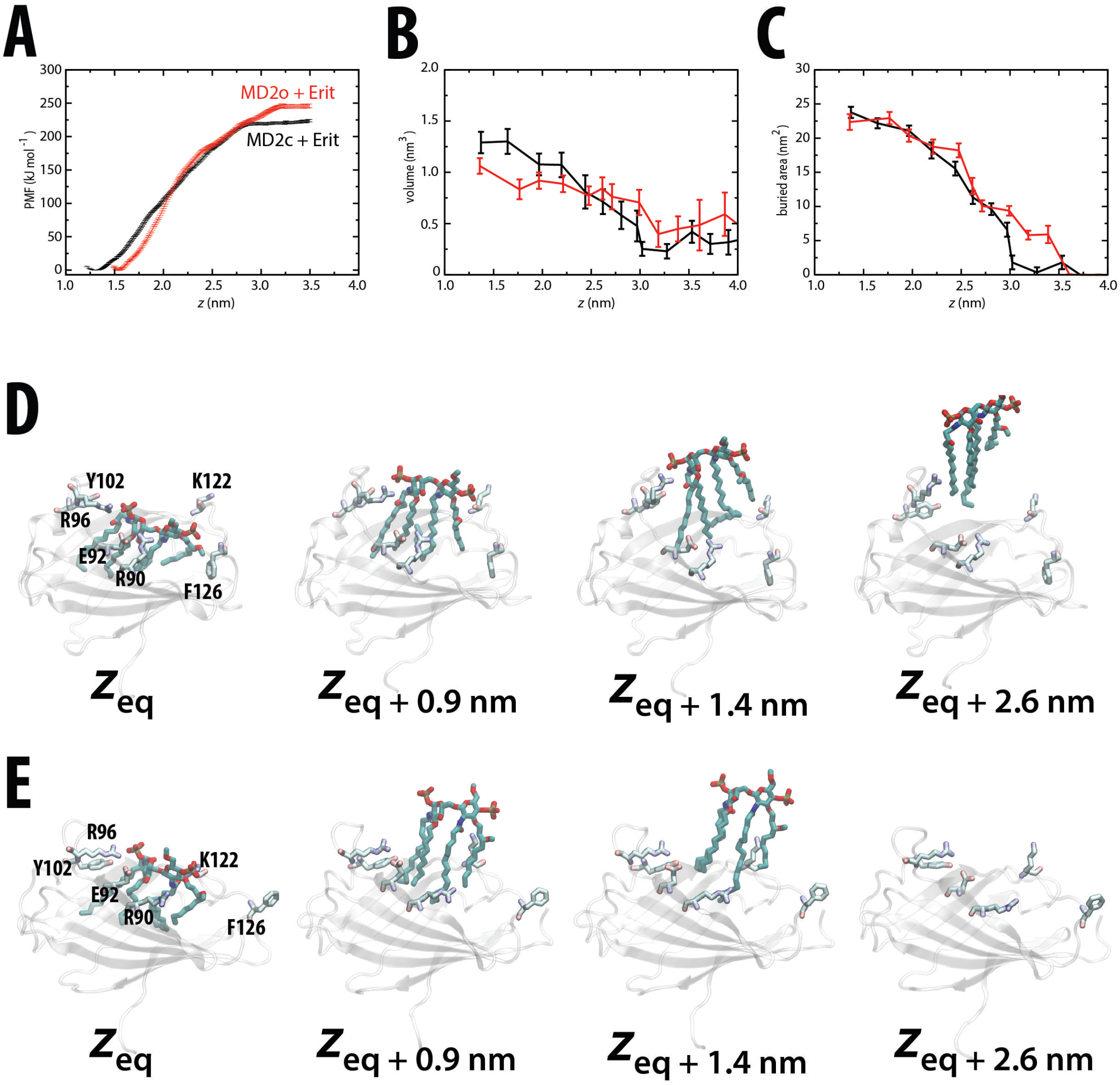

Figure S5. Eritoran PMF. **(A)** PMF curves calculated as a function of *z* for Eritoran binding to MD-2c (black line) and MD-2o (red line). The PMFs have been normalized so that the minimum lies at 0 kJ mol^{-1}. Sample standard deviation was estimated using 200 bootstraps over converged data. **(B)** Protein cavity volume and **(C)** buried surface area between protein and lipid, both shown as a function of PMFs for Eritoran binding to MD-2c (black line) and MD-2o (red line). Protein cavity volume was calculated using trj_cavity, and mean and standard deviations are shown for the final 5 ns of the corresponding PMF windows. Snapshots of Eritoran at different positions along *z* with respect to its equilibrium bound position (z_{eq}), are shown for **(D)** MD-2c and **(E)** MD-2o. The lipid molecule is shown in thick wireframe CPK format. Key side chains are labelled and shown in thin wireframe format, with the MD-2 fold rendered as transparent cartoons.

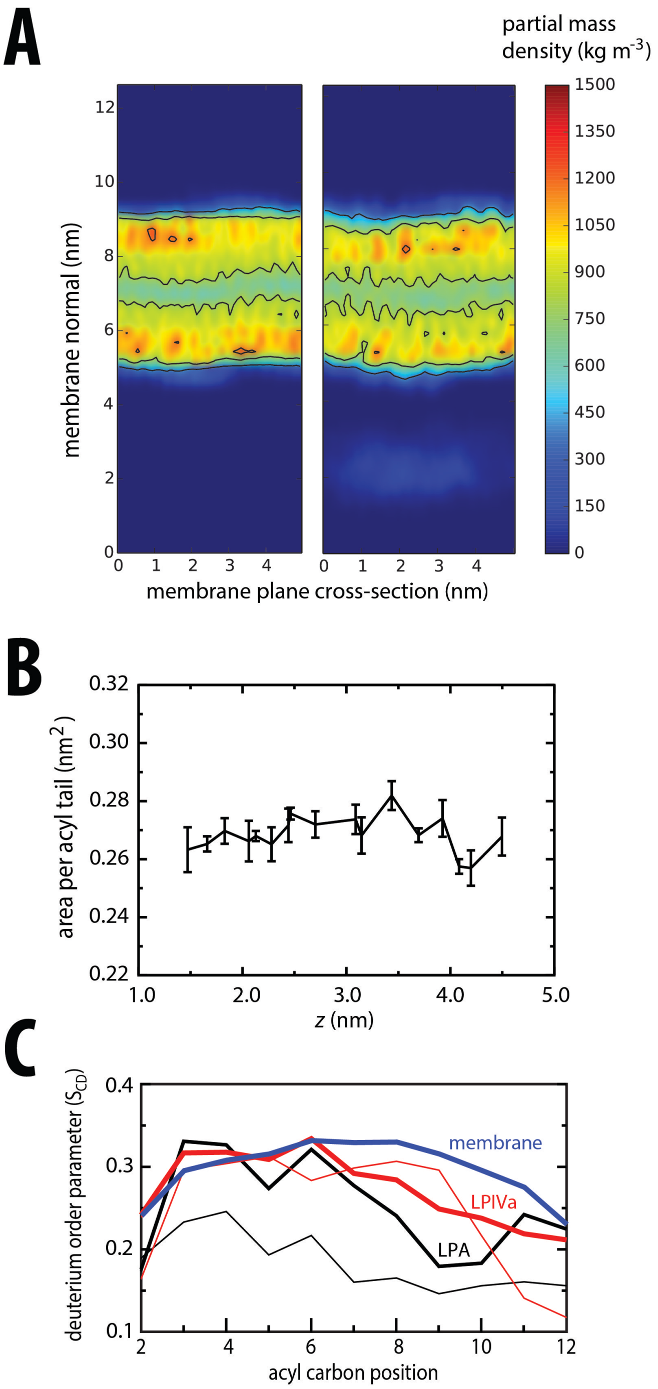

Figure S6. Dependence of lipid and membrane structure/dynamics upon PMF for extraction of LPA from symmetric LPA bilayer in presence of Mg^{2+}. **(A)** Contour map of lipid partial density for bilayer cross-section calculated along membrane normal, at equilibrium (left) and upon extraction of a single LPA molecule (right). **(B)** Mean area per acyl tail of bilayer, calculated as a function of PMF z. Mean and standard deviation are calculated over the final 5 ns of each window. **(C)** Deuterium order parameters, averaged across all acyl chains, calculated for LPA within a bilayer phase (blue), and for LPA (black) and LPIVa (red) at their equilibrium bound position z_{eq} in MD-2c (thick lines) and MD-2o (thin lines) protein conformations. In the dissolved state, the mean S_{CD} across all acyl tail carbons is <0.1 for both LPA and LPIVa. Order parameters were calculated with respect to the membrane normal, or in the case of protein, the cavity axis was defined as the reference vector.

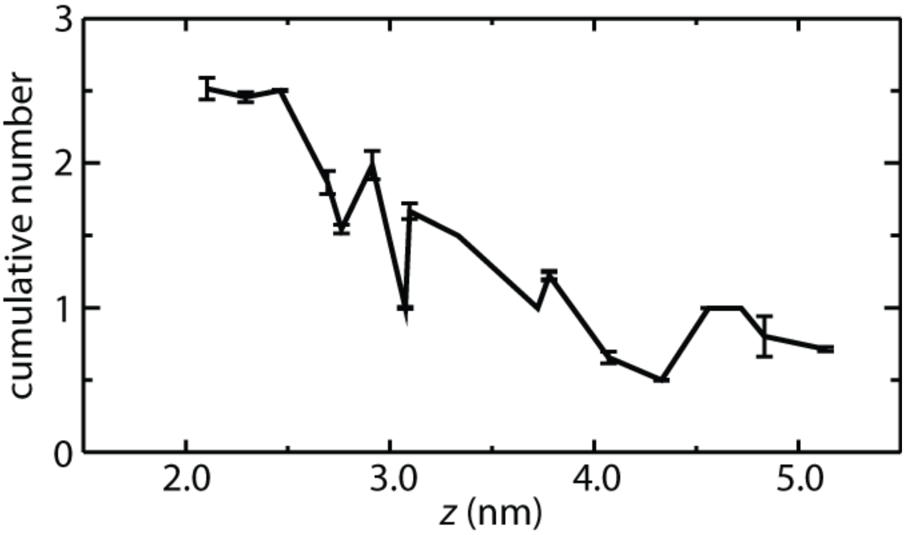

Figure S7. Cumulative number of Mg^{2+} ions within ≤ 1 nm of phosphate oxygens of single LPA molecule as a function of PMF *z* in membrane system. Means are calculated from RDFs over the final 5 ns of each window, with standard deviations estimated by calculating individual RDFs over five 1 ns windows.

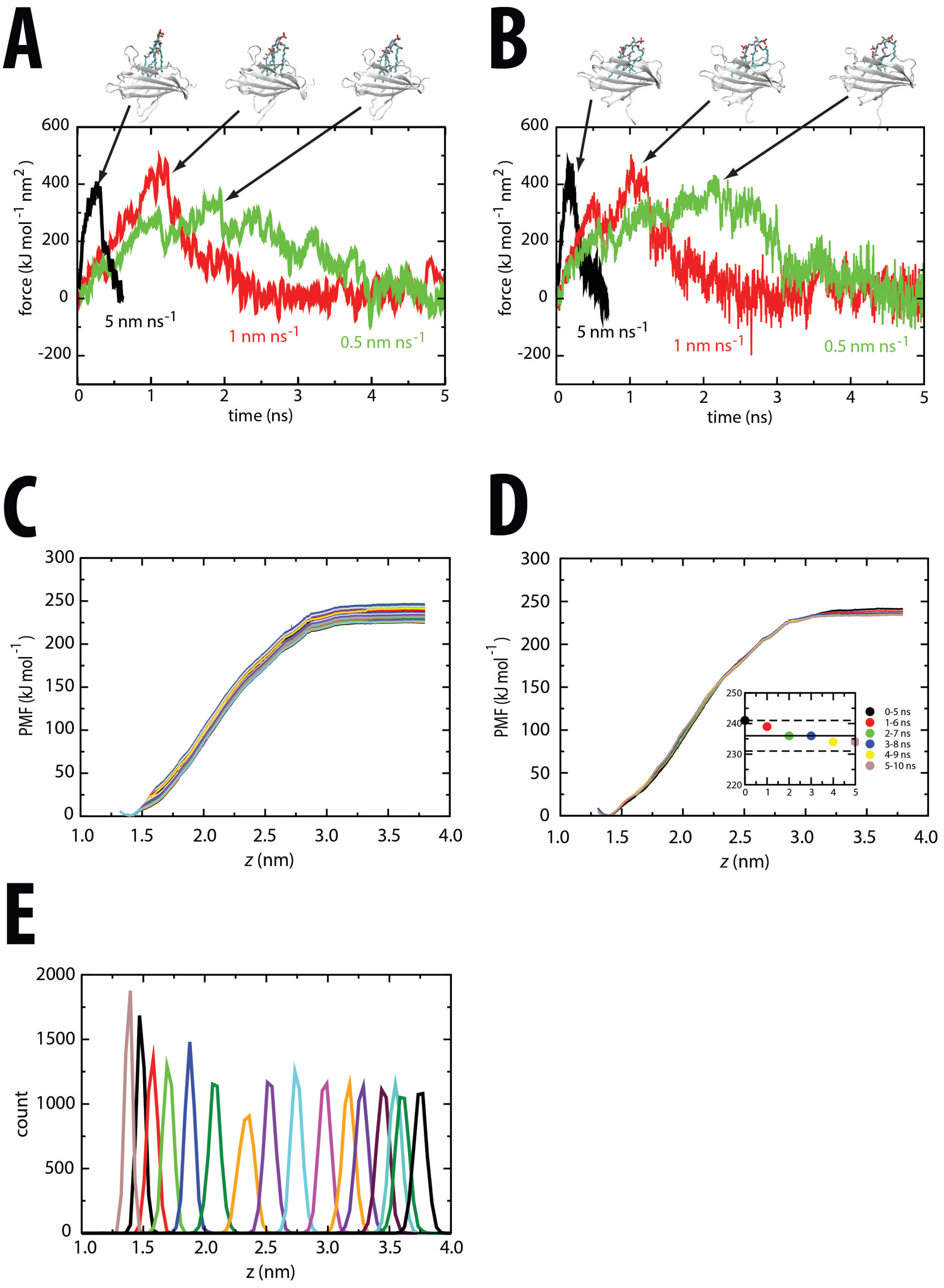

Figure S8. Convergence **of** steered MD and PMF calculations. Force versus time curves during steered MD simulations are shown for **(A)** MD-2c+LPIVa, and **(B)** MD-2o+LPIVa. Snapshots corresponding to the point of rupture (maximum pulling force) are shown above each graph. Data are shown for several independent pulling simulations, using spring velocities of 5 nm ns^{-1} (black), 1 nm ns^{-1} (red), and 0.5 nm ns^{-1} (green). Example convergence data are shown for MD-2o+LPIVa PMFs in terms of **(C)** boostrap WHAM profiles, **(D)** successive, overlapping 5 ns block profiles with average total free energies shown inset, and **(E)** unbiased umbrella sampling histograms.

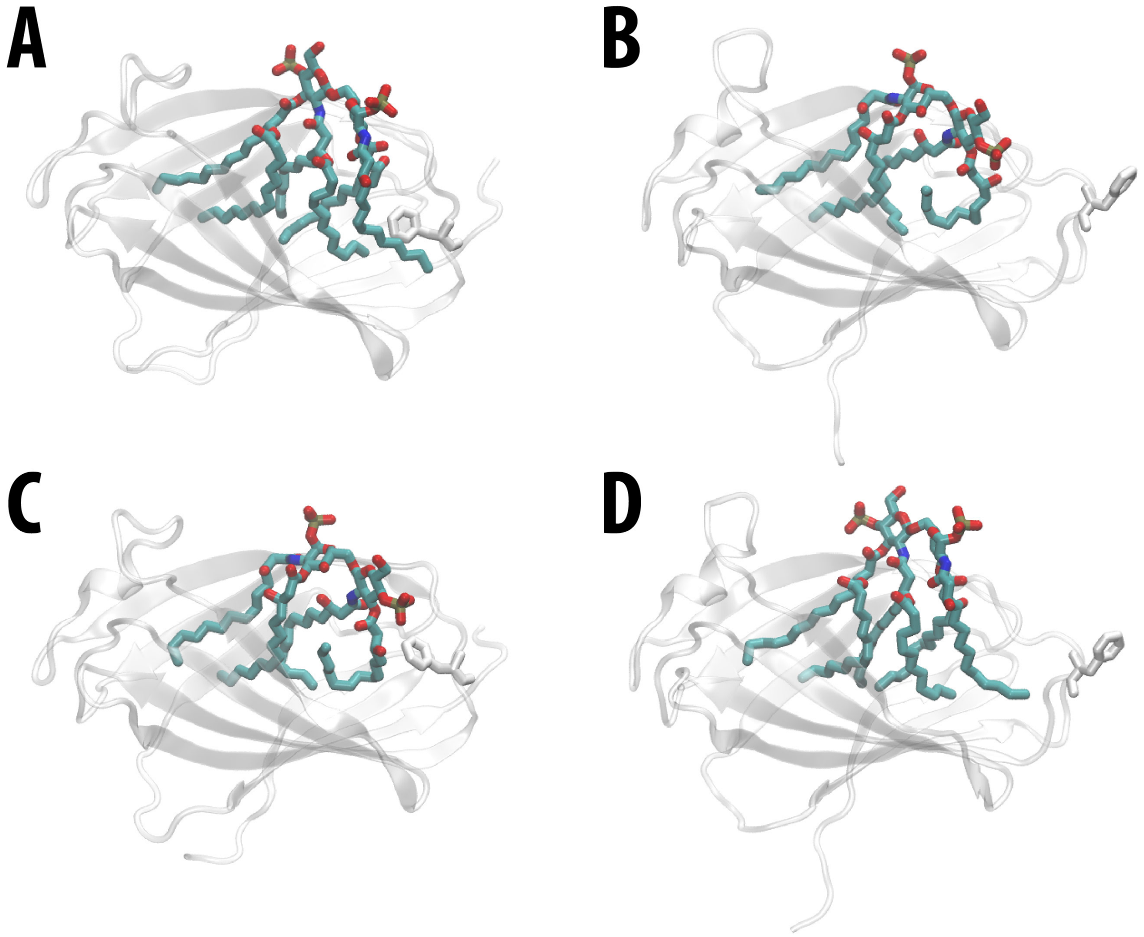

Figure S9. Setup of human MD-2 simulation systems. Snapshots are shown for **(A)** MD-2c bound to lipid A, obtained from the crystal structure of the active TLR4•MD-2 receptor complex bound to LPS[14] (pdb: 3FXI), and **(B)** the crystal structure of MD-2o bound to lipid IVa (pdb: 2E59). Snapshots of the corresponding "opposite" MD-2 configurations were obtained via pair-wise structural alignment and retention of ligand coordinates, as shown for **(C)** MD-2c bound to lipid IVa, and **(D)** MD-2o bound to lipid A.
